# The diagnostic yield of a structured comorbidity workup in a real-life outpatient population with obstructive sleep apnea: a cross-sectional study

**DOI:** 10.1080/07853890.2026.2622175

**Published:** 2026-02-04

**Authors:** Xiaolei Zhang, Huan Tang, Teng Han, Yiming Li, Linfan Su

**Affiliations:** a Department of Pulmonary and Critical Care Medicine, Center of Respiratory Medicine, China-Japan Friendship Hospital, Beijing, China; b National Clinical Research Center for Respiratory Diseases, Beijing, China; c The Graduate School of Peking Union Medical College, Chinese Academy of Medical Science and Peking Union Medical College, Beijing, China; d Capital medical university, Beijing, China; e State Key Laboratory of Respiratory Health and Multimorbidity, Beijing, China

**Keywords:** Obstructive sleep apnea, comorbidity, screening, cardiometabolic complication

## Abstract

**Background:**

Obstructive sleep apnea (OSA) is associated with multiple systemic complications. However, evidence-based recommendations for comorbidity screening remain sparse. This study aimed to evaluate whether a structured comorbidity workup could yield clinically meaningful diagnostic gains in OSA patients.

**Methods:**

In this single-center cross-sectional observational study, patients diagnosed with OSA underwent a standardized comorbidity assessment including laboratory tests, electrocardiogram, echocardiography, abdominal ultrasound, ambulatory blood pressure monitoring, spirometry, and validated questionnaires.

**Results:**

A total of 1904 OSA patients were included. Before evaluation, patients had a median of two [IQR 1–2] known comorbidities. The structured workup identified 3,200 newly diagnosed conditions, with a median of two [IQR 1–2] new diagnoses per patient. The most frequent comorbidities were dyslipidaemia (55%), type 2 diabetes mellitus (32%), metabolic syndrome (31%), hypertension (30%), non-alcoholic fatty liver disease(29%), hyperuricemia (29%), significant coronary artery disease (15%), arrhythmia (16%), heart failure with preserved ejection fraction (13%), obesity hypoventilation syndrome (19%), chronic obstructive pulmonary disease (8%) , asthma (12%), gastro-oesophageal reflux disease (17%), anxiety or depression (17%), and thyroid dysfunction (6%). Older age, male sex and lower mean oxygen saturation were independently associated with a higher comorbidity burden. Newly identified conditions led to pharmacological treatment changes in 40%, specialist referral in 54%, and lifestyle interventions in 85% of cases.

**Conclusion:**

A structured comorbidity workup in OSA patients reveals a high prevalence of previously unrecognized, clinically actionable conditions, particularly cardiometabolic disorders. Integrating systematic screening into routine OSA care may improve risk stratification, treatment optimization, and prevention of adverse outcomes (See Graphic Abstract).

## Graphical Abstract

**Figure:**
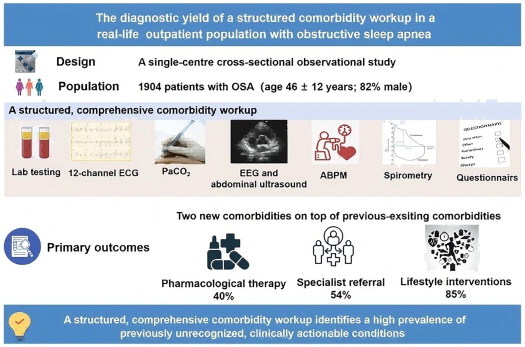

## Introduction

Obstructive sleep apnoea (OSA) is a highly prevalent sleep-related breathing disorder affecting an estimated 9-38% of the general adult population, with prevalence rising sharply with age, male sex, and obesity [1]. OSA represents a significant and growing challenge for global healthcare systems due to its rising prevalence, substantial public health impact, and increasing economic burden. Beyond its hallmark features of recurrent nocturnal upper-airway obstruction, intermittent hypoxaemia, and sleep fragmentation, OSA is increasingly recognized as a multi system disorder with far-reaching cardiovascular, metabolic, respiratory and neuropsychiatric consequences [2–6]. Epidemiological studies have consistently linked OSA to hypertension, atrial fibrillation, coronary artery disease, heart failure, type 2 diabetes, dyslipidaemia, chronic airway diseases and depression [7–11]. These comorbidities not only amplify the morbidity and mortality burden associated with OSA but also influence the management strategies and treatment adherence, particularly to continuous positive airway pressure (CPAP) therapy [12,13]. Timely identification and management of co-existing conditions are therefore integral to comprehensive OSA care and to reducing long-term cardiovascular risk. Current OSA guidelines emphasize the importance of managing comorbidities alongside symptom relief [3,14].

OSA is closely linked to a broad spectrum of cardiometabolic diseases; however, the real-world detection of these comorbidities in routine sleep clinic practice remains highly variable. In many clinical settings, comorbidity detection often relies on opportunistic assessment or patient-reported history, rather than on systematic and standardized screening. Consequently, a substantial proportion of clinically relevant cardiometabolic complications may remain unrecognized at the time of OSA diagnosis. To date, evidence quantifying the diagnostic yield of a structured comorbidity screening protocol in an unselected, real-world OSA outpatient population is limited. Most prior studies have relied on selected cohorts, retrospective analyses, or have focused on individual comorbidities rather than a comprehensive cardiometabolic assessment. To our knowledge, no prospective studies have systematically quantified the diagnostic yield of a structured comorbidity screening protocol in an unselected, real-world OSA clinic population. Addressing this gap is clinically important, as early identification of previously undiagnosed cardiometabolic complications may enable timely intervention, support integrated patient management, and improve long-term cardiovascular risk stratification.

In this study, we systematically quantified the diagnostic yield of a standardized comorbidity screening protocol in routine OSA clinical practice. We sought to (1) determine the number and types of previously undiagnosed comorbidities identified by a protocolized workup in adults with OSA, and (2) assess the proportion of these findings that were actionable in terms of guideline-directed management. Our aim was to testify whether a structured workup for comorbidity could yield clinically meaningful diagnostic gains in OSA, thereby providing clinicians with actionable insights to refine strategies for screening and managing OSA patients.

## Methods

### Study design and population

We conducted a single-centre, cross-sectional observational study at the sleep disorders center in China Japan friendship hospital, Beijing, China, between November 2022 and April 2025. Consecutive adults (20–75 years) referred to our outpatient clinic for suspective evaluation of OSA, mostly by medical specialists from our hospital, general practitioners or surrounding hospitals were enrolled. Inclusion criteria included patients diagnosed with OSA during the workup, defined by an apnoea-hypopnoea index (AHI) >5 events/h on polysomnography (PSG) or home sleep apnoea testing (HSAT), in accordance with international criteria [15]. Full PSG examination was performed with the NoxA1 device (Nox Medical, Inc., Reykjavik, Iceland). The PSG setup included a recording of electroencephalography (EEG), electrooculography (EOG), chin electromyography (EMG), nasal pressure, snoring, rib cage and abdominal movement, pulse oximetry, activity, and body position. The EEG was recorded with six channels (F4, C4, and O2 with the F3, C3, and O1 channels as backup), and the EOG was recorded with two channels (E1 and E2). The chin EMG was recorded with two electrodes placed below the mandible, and one reference electrode placed above the mandible. HSAT was conducted by the overnight home use of Nox-T3 monitor (Nox Medical, Inc., Reykjavik, Iceland) that recorded nasal pressure, snoring, rib cage and abdominal movement, pulse oximetry, activity, and body position. The manual scoring was performed by an experienced sleep technician according to the AASM manual. Patients with central sleep apnea, recent (<90 days) hospitalization, pregnancy or inability to complete the diagnostic workup were excluded. All patients provided written informed consent before the workup at our outpatient clinic. The study was approved by the ethics committee of China Japan friendship hospital. We followed the EQUATOR guidelines for reporting, using the STROBE checklist for observational research (Supplementary File).

### Baseline assessment

At enrollment all participants underwent a standardized baseline assessment that included a structured medical history, medication inventory, and review of the electronic health record. The number of prior comorbidities referred to conditions that were previously diagnosed and documented before the OSA diagnostic workup. Known comorbidities were identified through extraction from the electronic health record and were subsequently confirmed during patient interviews to ensure accuracy.

### Protocolized comorbidity workup

Each patient completed a predefined battery of investigations targeting common cardiovascular, metabolic, neuropsychiatric, and respiratory comorbidities of OSA. Diagnostic tests were not repeated if already performed within 6 months before the baseline visit in our medical system.

The protocol included:

1. Anthropometrics: body mass index (BMI)
2. Laboratory testing: A blood sample was obtained to assess total blood count, high-sensitivity C-reactive protein (hs-CRP), cholesterol (total, LDL, HDL), triglycerides, fasting plasma glucose(FPG), oral glucose tolerance test (OGTT), haemoglobin A1c (HbA1c), alanine aminotransferase (ALT), aspartate aminotransferase (AST), uric acid, creatinine, thyroid-stimulating hormone, free T4,N-terminal pro-B-type natri-ureticpeptide(NT-proBNP)
3. Arterial partial pressure of carbon dioxide (PaCO_2_): Blood gas analysis for patients with BMI more than 30 kg/m^2^
4. Twelve-lead electrocardiogram (ECG) at rest
5. Transthoracic echocardiography at rest and abdominal ultrasound
6. Twenty-four hours ambulatory blood pressure monitoring
7. Pulmonary function testing: Spirometry in current/former smokers, patients with chronic respiratory symptoms or occupational exposure history
8. Screening for CAD was not routine and coronary imaging evaluation was conducted only when indicated by symptoms or abnormal ECG manifestations
9. Neuropsychiatric questionnaires: Epworth Sleepiness Scale (ESS), PHQ-9 (Patient Health Questionnaire-9), Generalised Anxiety Disorder-7 (GAD-7)
10. Symptom-based evaluation of Gastro-oesophageal reflux disease (GERD Questionnaire)

Each patient’s diagnostic results were evaluated by the treating physician in collaboration with a multidisciplinary team. By integrating all available data, the team reached a consensus on the most accurate diagnosis and preferred treatment strategy. The treating physician communicated the recommended plan, including any additional testing, to the patient to support shared decision-making and documented new diagnoses and treatment plans in the electronic health record.

### Definitions of new comorbidity

A comorbidity was classified as “newly diagnosed” if it was not documented in the medical record or patient-reported history before enrolment, and it met accepted diagnostic criteria during the study workup, which may resulted in initiation, change, or reinforcement of treatment, or prompted referral for specialist care. Hypertension, type 2 diabetes mellitus (T2DM), dyslipidemia, cardiac arrhythmias (atrial fibrillation, ventricular tachyarrhythmias and bradyarrhythmia), coronary artery disease (CAD), heart Failure with preserved ejection fraction (HFpEF), non-alcoholic fatty liver disease (NAFLD), metabolic syndrome, hyperuricemia, obesity hypoventilation syndrom (OHS), thyroid dysfunction, depression, anxiety, chronic obstructive pulmonary disease (COPD), asthma and gastro-oesophageal Reflux Disease (GERD) were defined using standard clinical and guideline-based diagnostic criteria [16–22].

### Sample size estimation

The sample size was calculated based on estimation of a proportion in a cross-sectional study. Assuming an expected prevalence (p) of cardiometabolic complications among OSA patients of 0.5, a two-sided significance level of 0.05, and an allowable absolute error (d) of 0.05, the minimum required sample size was calculated using the following formula:

n=Zα22⋅p(1−p)d2

After accounting for an anticipated 10% rate of incomplete or invalid data, a total of at least 430 participants were required for inclusion in the study. The calculated sample size represented the minimum number of participants required. During the study period, a larger number of eligible patients were consecutively recruited, and all eligible participants were included in the final analysis to improve the precision of prevalence estimates.

### Statistical analysis

Analyses were performed on participants with complete data for the variables of interest. Sensitivity analyses were performed using complete-case data to assess the robustness of the primary results. Quantitative variables were assessed for normality using the Shapiro-Wilk test. Normally distributed variables were summarized as mean ± standard deviation, and non-normally distributed variables as median (interquartile range). Skewed variables were log-transformed prior to inclusion in multivariable models. The diagnostic yield was expressed as the median [interquartile range (IQR)] number of new comorbidities per patient. Associations between patient characteristics and diagnostic yield were assessed using multivariable linear regression or logistic regression as appropriate. Analyses were performed using SPSS 25 (IBM), with *p* < 0.05 considered statistically significant. Several measures were taken to address potential sources of bias. To minimize selection bias, consecutive eligible participants were recruited during the study period. Information bias was reduced by using standardized data collection procedures and predefined diagnostic criteria. Recalling bias was minimized by extracting relevant clinical information from medical records whenever possible. Potential confounders, including age and sex, were identified a priori and adjusted for in the statistical analyses.

## Results

### Study population

**Figure 1.**
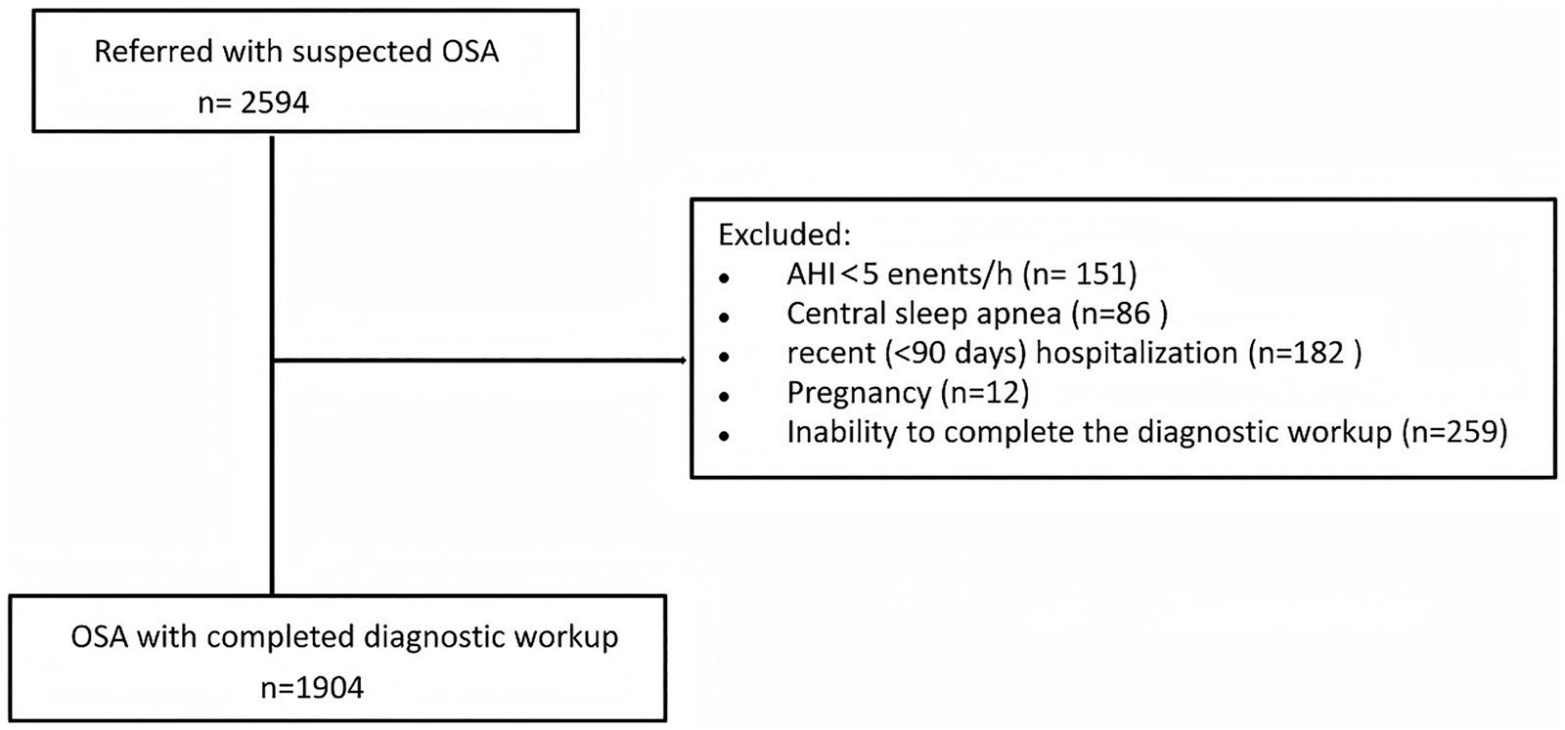
Flowchart of participants in this study. OSA: obstructive sleep apnea; AHI: apnea hypopnea index.

**Figure 2.**
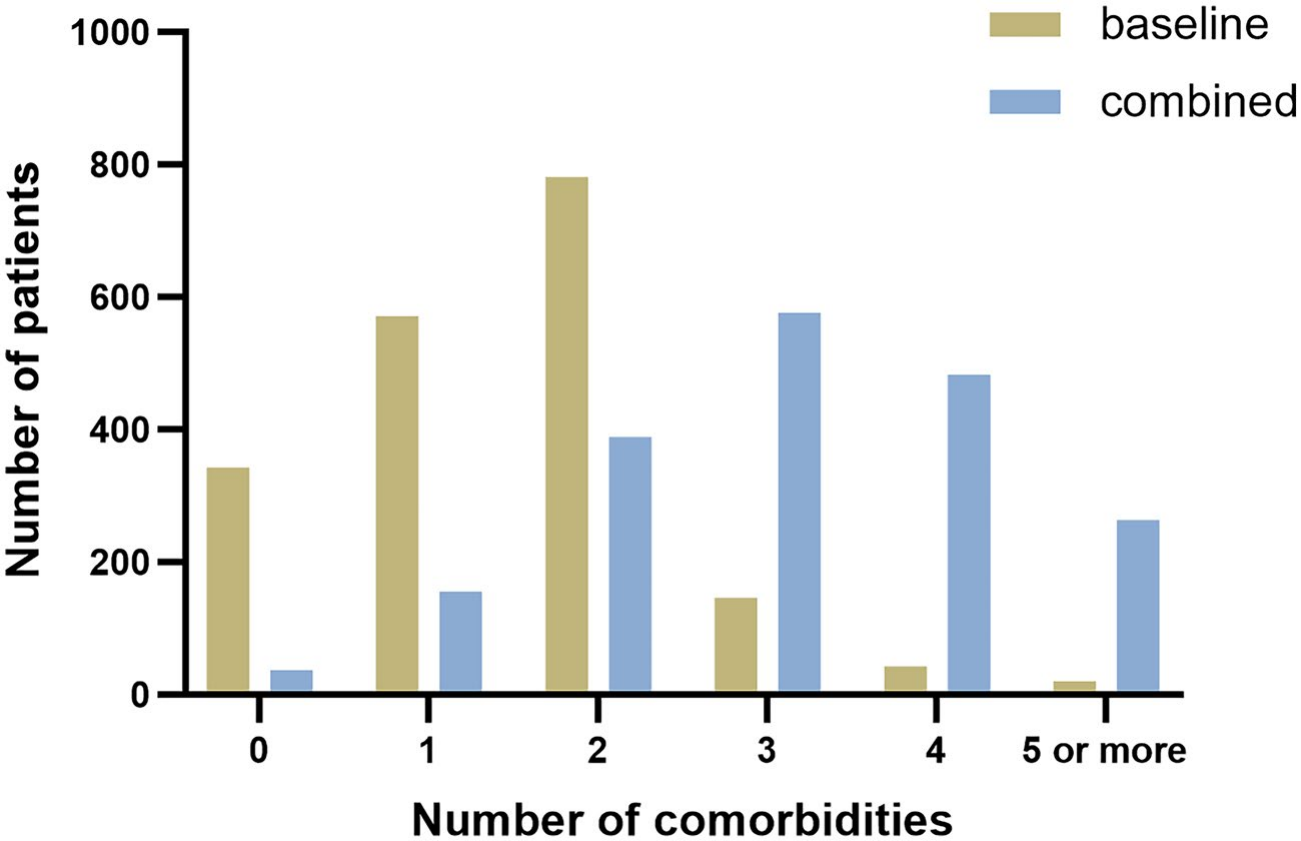
Number of baseline and combined comorbidities.

A total of 1904 patients with OSA were enrolled and the mean age was 46 ± 12 years, 82% were male, and the median body mass index (BMI) was 28.7 kg/m^2^ (Figure 1). The median apnoea hypopnea index (AHI) was 33.2 events·h^−1^ (IQR [14.6–59.2]), with 26% classified as mild, 20% moderate, and 54% severe OSA. At baseline, prior to the diagnostic workup, patients presented with a medical history of a median of two [IQR 1–2] comorbidities. Three hundred forty-three (18%) patients had no comorbidity, 571 (30%) had one comorbidity, 781 (41%) had two comorbidities and 209 (11%) had three or more comorbidities (Table 1, Figure 2).

**Table 1.** Baseline characteristics.

Characteristic	Total (*n* = 1904)
Age, yrs	46 ± 12
Male, n (%)	1575 (82%)
BMI, kg/m²	28.7 ± 4.4
Current smoker,%	17%
ESS	9.8 ± 4.1
hs-CRP, mg/L	1.72 (0.90, 2.96)
TST,min	417 ± 82
Sleep efficiency,%	82.7 ± 12.7
N1,%	9.6 (5.5, 17.0)
N2,%	39.6 (31.2, 47.1)
N3,%	12.3 (5.1, 18.5)
REM,%	17.2 (13.1, 21.5)
AHI,	33.2 [14.6–59.2]
AI	14.6 (3.6, 45.4)
HI	10.1 (5.4, 17.5)
Mid OSA, n (%)	501 (26)
Moderate OSA, n (%)	385 (20)
Severe OSA, n (%)	1040 (54)
ODI	31.0 (10.9, 63.4)
SpO_2_baseline	95.3 ± 4.8
SpO_2_mean	93.3 ± 5.9
LSpO_2_	75.0 ± 15.7
T90	5.4%(1.3%, 27%)

*Notes:* Data are presented as number (%), mean ± standard deviation, or median [interquartile range].

BMI: body mass index; ESS: Epworth Sleepiness Scale; hs-CRP: high-sensitivity C-reactive protein; TST: total sleep time; REM: rapid eye movement; AHI: apnea hypopnea index; AI: apnea index; HI: hypopnea index; OSA: obstructive sleep apnea; ODI: oxygen desaturation index; SpO_2_: pulse oxygen saturation; LSpO_2_: lowest pulse oxygen saturation; T90: time with oxygen saturation below 90%.

### Diagnostic yield

**Figure 3.**
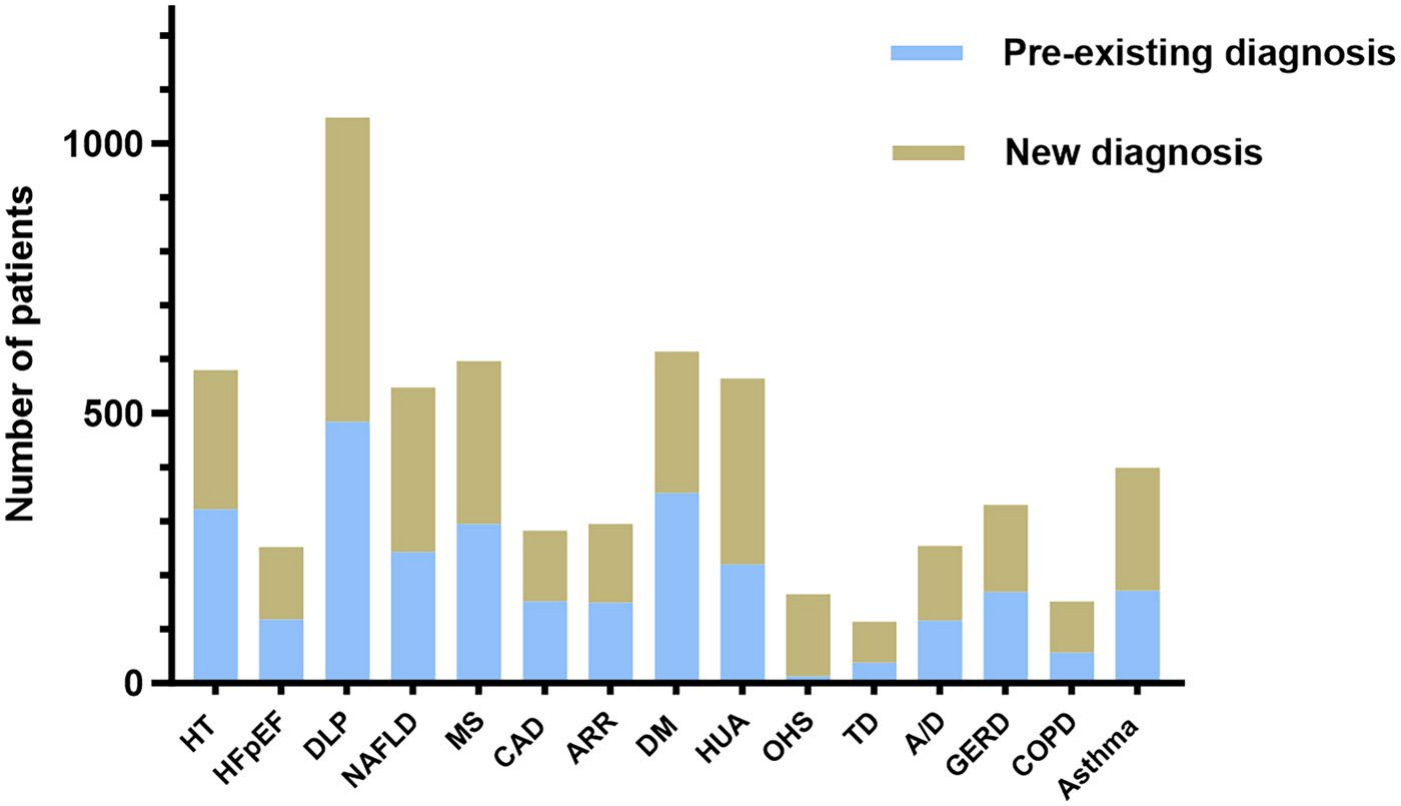
Number of pre-existing and new diagnosis. HT: hypertension; HFpEF: heart Failure with preserved ejection fraction; DLP: dyslipidemia; MS: metabolic syndrome; ARR: arrhythmia; HUA: hyperuricemia; T2DM: type 2 diabetes mellitus; OHS: obesity hypoventilation syndrome; TD: thyroid dysfunction; A/D: anxiety or depression; GERD: gastro-oesophageal Reflux Disease; COPD: chronic obstructive pulmonary disease.

The protocolized workup identified a total of 3,200 newly diagnosed comorbidities, with a median of two (IQR 1–2) new diagnoses per patient besides OSA. Seven hundred and four patients (37%) had 1 new comorbidity, 724 (38%) had 2 new comorbidities, 305 (16%) had 3 new comorbidities, 57 (3%) had more than 4 or more comorbidities and only 114 (6%) patients did not get any new diagnoses (supplement flowchart illustrating the distribution of baseline versus newly detected comorbidities stratified by OSA severity). The most frequent new diagnoses were hypertension (258, 13%), dyslipidaemia (563,30%), NAFLD (304,16%), metabolic syndrome (301,16%), and T2DM (261,14%), hyperuricemia (344, 18%). Other important comorbidities that need to initiate prompt clinical intervention were HFpEF (135,7%), significant CAD (131,7%), arrhythmia (146,8%), OHS (152,8%), COPD (95,5%), asthma (133.7%), GERD (162,8%) and anxiety or depression (139,7%) (Table 2, Figure 3).

**Table 2.** Pre-existing and newly diagnosed commodities in patients with OSA.

Diagnosis	Pre-existing diagnosis	New diagnosis	Total diagnosis
Hypertension, n (%)	322 (17%)	258 (13%)	580 (30%)
HFpEF, n(%)	118 (6%)	135 (7%)	253 (13%)
Dyslipidaemia, n (%)	485 (25%)	563 (30%)	1048 (55%)
NAFLD	243 (13%)	304 (16%)	547 (29%)
Metabolic syndrome	295 (15%)	301 (16%)	596 (31%)
Signifcant CAD, n (%)	152 (8%)	131 (7%)	283 (15%)
arrhythmia, n (%)	149 (8%)	146 (8%)	295 (16%)
T2DM, n (%)	353 (18%)	261 (14%)	614 (32%)
hyperuricemia	220 (11%)	344 (18%)	564 (29%)
OHS, n (%)	13 (1%)	152 (8%)	165 (9%)
Thyroid dysfunction, n (%)	38 (2%)	76 (4%)	114 (6%)
Anxiety or depression	116 (6%)	139 (7%)	255 (13%)
GERD	169 (9%)	162 (8%)	331 (17%)
COPD	57 (3%)	95 (5%)	152 (8%)
Asthma	106 (6%)	133 (7%)	248 (13%)

*Notes:* HFpEF: heart Failure with preserved ejection fraction; NAFLD: non-alcoholic fatty liver disease; CAD: coronary artery disease; T2DM: type 2 diabetes mellitus; OHS: obesity hypoventilation syndrom; GERD: gastro-oesophageal Reflux Disease; COPD: chronic obstructive pulmonary disease.

Combining the comorbidities at baseline with the newly diagnosed comorbidities, we observed that patients with OSA in total have a median of 3 [IQR 2–4] comorbidities besides OSA. One hundred fifty-five patients (8%) had one comorbidity, 389 (20%) had 2 comorbidities, 576 (30%) had 3 comorbidities, 483 (25%) had 4 comorbidities, 264 (14%) had 5 or more comorbidities and only 37 (2%) patients did not have any comorbidity. The common comorbidities are hypertension (580, 30%), dyslipidaemia (1048, 55%), NAFLD(547, 29%), metabolic syndrome (596,31%), and T2DM (614,32%), hyperuricemia (564, 29%), HFpEF (253,13%), significant CAD (283,15%), arrhythmia (295,16%), OHS (165,9%), COPD (152,8%) and anxiety or depression (331,17%), GERD (331,17%), asthma (228,12%), and thyroid dysfunction (114,6%), (Table 2, Figure 2).

### Predictors of diagnostic yield and factors associated with the prevalence of common cardiometabolic comorbidities

In multivariable regression analysis, older age, male sex and mean pulse oxygen saturation (SpO_2_mean) were independently associated with a greater number of total comorbidities. BMI, total sleep time (TST), sleep structure parameters, AHI and high sensitive C-reactive protein (hs-CRP) were not significant predictors. We observed no significant associations between the number of newly diagnosed comorbidities and the severity of OSA or anthropomorphic parameters except for age (Table 3).

**Table 3.** Regression analysis of common cardiometabolic comorbidities.

	Age (Beta, P)	Male sex (Beta, P)	BMI (Beta, P)	Current smoker (Beta, P)	ESS (Beta, P)	TST (Beta, P)	SE (Beta, P)	AHI (Beta, P)	N3% (Beta, P)	ODI (Beta, P)	SpO2mean(Beta, P)	T90 (Beta, P)	hs-CRP (Beta, P)
Number of totle comorbidities	0.153 <0.001	0.082 0.047	0.017 0.661	0.056 0.451	0.037 0.546	−0.071 0.205	0.029 0.659	0.125 0.357	0.073 0.126	0.291 0.054	−0.204 0.009	0.009 0.919	0.031 0.424
Number of newly diagnosed comorbidities	0.139 <0.001	0.027 0.507	−0.028 0.475	0.023 0.551	0.045 0.232	0.004 0.911	0.012 0.772	−0.069 0.077	0.074 0.058	−0.053 0.172	0.001 0.984	−0.031 0.420	0.029 0.457

*Notes:* BMI: body mass index; ESS: Epworth Sleepiness Scale; hs-CRP: high-sensitivity C-reactive protein; TST: total sleep time; AHI: apnea hypopnea index; ODI: oxygen desaturation index; SpO_2_: pulse oxygen saturation; T90: time with oxygen saturation below 90%.

As for the factors associated with the prevalence of individual cardiometabolic comorbidity of OSA, age, TST, ODI and SpO_2_mean were independently associated with the prevalence of metabolic syndrome; age and ODI were associated with the prevalence of T2DM; age, N3% and ODI were associated with the prevalence of dyslipidemia; age, TST and T90 were associated with the prevalence of NAFLD. No significant associations between SDB parameters and the prevalence of hypertension or hyperuricemia (Table 4).

**Table 4.** Regression analysis of common cardiometabolic comorbidities.

	Hypertension (Beta, *P*)	Metabolic syndrom (Beta, *P*)	HFpEF (Beta, *P*)	CAD (Beta, *P*)	Arrhythmia (Beta, P)	T2DM (Beta, *P*)	Dyslipidemia (Beta, *P*)	NAFLD (Beta, *P*)	hyperuricemia (Beta, *P*)
Age	0.050,<0.001	0.043, <0.001	0.011, 0.040	0.091,<0.001	0.056, 0.005	0.046, <0.001	−0.022, 0.009	−0.028, 0.001	−0.041, <0.001
Male sex	0.235, 0.346	0.281, 0.259	0.024, 0.547	0.776, 0.072	0.432, 0.403	0.216, 0.363	0.433, 0.078	0.727, 0.018	1.478, <0.001
BMI	0.008, 0.272	0.008, 0.252	0.057, 0.138	0.004, 0.871	0.005, 0.760	0.004, 0.601	0.002, 0.729	0.001, 0.867	−0.004, 0.664
Current smoker	0.327, 0.092	0.007, 0.482	0.009, 0.754	0.029, 0.075	0.008, 0.683	0.010, 0.31	0.008, 0.421	0.007, 0.435	0.023, 0.636
ESS	0.007, 0.308	0.076, 0.001	0.022, 0.828	0.047, 0.056	0.013, 0.293	0.004, 0.483	0.009, 0.172	0.001, 0.978	0.015, 0.017
TST	−0.001, 0.641	−0.004, 0.019	−0.002, 0.043	0.003, 0.379	0.001, 0.796	0.002, 0.319	0.001, 0.873	−0.003, 0.046	0.016, , 0.340
SE	−0.008, 0.447	−0.040, 0.749	0.029, 0.591	−0.008, 0.696	0.004, 0.875	0.002, 0.882	−0.008, 0.504	0.014, 0.199	0.018, 0.091
AHI	−0.066, 0.719	−0.050, 0.779	0.017,<0.001	0.014, 0.532	0.012, 0.680	−0.076, 0.661	0.004, 0.756	−0.006, 0.644	−0.002, 0.830
N3%	0.014, 0.259	0.008, 0.537	−0.039, 0.318	−0.001, 0.966	−0.028, 0.363	−0.013, 0.295	−0.033, 0.017	0.024, 0.066	0.001, 0.948
ODI	0.007, 0.366	0.015, 0.036	0.125, 0.346	0.003, 0.904	0.025, 0.372	0.011, 0.083	0.032, <0.001	0.003, 0.779	0.002, 0.828
SpO_2_mean	−0.065, 0.146	−0.093, 0.036	−0.067, 0.186	0.342, 0.144	0.083, 0.021	−0.033, 0.451	0.006, 0.906	−0.009, 0.832	−0.043, 0.340
T90	−1.110, 0.251	−1.443, 0.124	0.091, 0.099	1.110, 0.664	1.950, 0.495	−1.245, 0.177	0.598, 0.596	1.665, 0.041	0.930, 0.330
hs-CRP	0.015, 0.429	0.008, 0.645	0.010, 0.798	0.067, 0.400	0.049, 0.615	0.028, 0.204	−0.007, 0.706	0.010, 0.586	0.016, 0.340

*Notes:* HFpEF: heart Failure with preserved ejection fraction; NAFLD: non-alcoholic fatty liver disease; CAD: coronary artery disease; T2DM: type 2 diabetes mellitus; OHS: obesity hypoventilation syndrom; GERD: gastro-oesophageal Reflux Disease; COPD: chronic obstructive pulmonary disease; BMI: body mass index; ESS: Epworth Sleepiness Scale; hs-CRP: high-sensitivity C-reactive protein; TST: total sleep time; AHI: apnea hypopnea index; ODI: oxygen desaturation index; SpO_2_: pulse oxygen saturation; T90 : time with oxygen saturation below 90%.

### Actionability, safety and feasibility

Of all newly diagnosed comorbidities, 40% prompted initiation or adjustment of pharmacological therapy, 54% led to specialist referral, and 85% resulted in lifestyle or behavioral interventions (Table 5). No adverse events occurred during testing procedures. The median time to complete the full workup was three days from enrolment, with 88% of patients completing all planned assessments.

**Table 5.** Actionability of new comorbidities.

New diagnosis	Initiation/adjustment of pharmacological therapy (n, %)	Specialist referral (n,%)	Lifestyle or behavioral interventions (n,%)
Hypertension	93 (36%)	199 (77%)	237 (92%)
Hypercholesterolaemia	155 (29%)	187 (35%)	535 (100%)
NAFLD	125 (41%)	170 (56%)	304 (100%)
metabolic syndrome	115 (39%)	124 (42%)	295 (100%)
Signifcant CAD	90 (68%)	133 (100%)	115 (87%)
Arrhythmia	53 (36%)	120 (82%)	55 (38%)
T2DM	154 (59%)	178 (68%)	261 (100%)
HfpEF	85 (63%)	103 (76%)	115 (85%)
Hyperuricemia	46 (21%)	62 (28%)	220 (100%)
OHS	23 (15%)	58 (38%)	152 (100%)
Thyroid dysfunction	21 (27%)	32 (42%)	28 (37%)
Anxiety or depression	23 (23%)	75 (65%)	61 (53%)
GERD	75 (46%)	78 (48%)	117 (72%)
COPD	26 (68%)	25 (67%)	21 (56%)
Asthma	108 (63%)	87 (51%)	60 (35%)

*Notes:* HFpEF: heart Failure with preserved ejection fraction; NAFLD: non-alcoholic fatty liver disease; CAD: coronary artery disease; T2DM: type 2 diabetes mellitus; OHS: obesity hypoventilation syndrom; GERD: gastro-oesophageal Reflux Disease; COPD: chronic obstructive pulmonary disease.

## Discussion

Patients with OSA often have multiple- comorbidities that impact their prognosis, yet the efficacy of comprehensive comorbidity screening in this group is not well-established. In this real-life outpatient cohort of patients with OSA, a structured comorbidity workup revealed a substantial burden of previously unrecognized conditions. Fifty seven percent of patients had at least two new diagnoses, and the average yield was two [IQR 1–2] newly detected comorbidities per patient. Importantly, the majority of these new findings were clinically actionable, prompting pharmacological initiation or adjustment, specialist referral, or targeted lifestyle counselling. Our findings provide compelling evidence for integrating routine comorbidity screening into the diagnostic workflow for OSA.

OSA is characterized by intermittent hypoxia, sleep fragmentation, and marked intrathoracic pressure swings, resulting in oxidative stress activation, sustained sympathoexcitation, hypothalamic-pituitary-adrenal (HPA) axis dysregulation, and elevated cardiac preload/afterload. These perturbations drive elevated blood pressure, metabolic dysregulation, systemic inflammation, endothelial dysfunction, and cardiac impairment, thereby increasing the risk of type 2 diabetes, coronary artery disease, cerebrovascular events, arrhythmias, and heart failure [23]. Consistent with previous reports from sleep clinic populations, our approach specifically uncovered a high prevalence and insufficient treatment of previously undiagnosed cardiometabolic complications, which further verify the high disease burden of OSA [24].

The under-diagnostic rate of cardio-metabolic complications for OSA remains poorly quantified in the literature. Several sleep clinic cohorts had reported that the prevalence of masked hypertension among OSA patients is about 34%, approximately 30% of T2DM cases in OSA are newly diagnosed, and about 20-30% of arrhythmia are newly identified at the time of sleep study [25–27]. Our newly detected diagnostic yield appears higher than that seen in registry-based retrospective analyses. In our cohort, more than half of patients with hypertension were newly diagnosed, possible reasons are because that nocturnal hypertension is especially common among OSA and relying solely on office BP may lead to significant underdiagnosis, which indicate that ABPM is crucial for detecting true hypertension burden in OSA. As for other cardio-metabolic complications, the new diagnostic yield is all near 50% percent. This may reflect both the comprehensive nature of our screening protocol and the fact that many OSA patients are managed in fragmented care settings where comorbidities may remain under-diagnosed. Currently, OSA patients are often managed in pulmonary or sleep clinics without comprehensive cardio-metabolic assessment, and most symptoms, such as fatigue, sleepiness, and dyspnea are unspecific and physicians may attribute these symptoms solely to aging, obesity or lack of exercise, overlooking formal cardio-metabolic risk evaluation, especially for non-obesity patients.

Screening for mood disorders is recommended in OSA patients, especially those with persistent symptoms despite treatment. Depression and anxiety in OSA are associated with reduced adherence to CPAP therapy, poorer quality of life, and increased cardiovascular morbidity. It is reported that the prevalence of depression among OSA patients is estimated at 20-45%, and anxiety prevalence varies more widely, from 11% to 70%, depending on the diagnostic methods used [28–30]. Both disorders are significantly more common in OSA populations than in the general public and frequently co-exist. Anxiety and depression are frequently underdiagnosed in patients with OSA. Studies reported that 30-60% of clinically significant mood disorders in OSA patients are not identified during routine clinical evaluations [31,32]. In our cohort, the prevalence of depression and anxiety are relatively low, possible reasons may be because patients may minimize or under-report symptoms of depression or anxiety, attributing them to lifestyle stress or poor sleep. Also, the lower self-rated psychological distress may be related to the social-culture background and differential item functioning for some questionnaires items across cultures. Culturally adapted instruments with clear confidentiality may improve psychological assessment in Asian populations.

Coupled interactions in biomechanics, airway inflammation, and neural reflexes occur between the upper and lower airways. Chronic airway disease, including asthma and COPD frequently co-occur with OSA, amplifying sleep fragmentation and ventilatory instability. Unrecognized diseases in airways or lungs may worsen nocturnal symptoms and oxygen desaturation, impair daytime function, increase the risk of acute exacerbation, contribute to poor positive airway pressure (PAP) tolerance and elevate all-cause and cardiovascular morbidity [33,34]. In our cohort, the prevalence of chronic airway disease among OSA is similar to previous studies. However, given the relatively greater complexity of spirometry and lower prevalence of chronic airway disease among OSA, we recommend conducting spirometry only in those who have unexplained respiratory symptoms or have an elevated risk profile based on their occupation or smoking history.

OHS is frequently under-diagnosed in patients with OSA. It is estimated that 70-90% of OHS cases remain unrecognized at the time of OSA diagnosis, which is in line with our study [33]. Many patients are classified as having ‘OSA with obesity’ without further evaluation of daytime hypercapnia. Undiagnosed OHS is associated with worse outcomes than OSA alone, including higher risks of pulmonary hypertension, heart failure, frequent hospitalizations, and increased mortality. Early recognition is crucial, as positive airway pressure (PAP) therapy and weight reduction interventions can substantially improve prognosis and quality of life. Therefore, routine evaluation of daytime or nocturnal hypercapnia should be integrated into clinical practice for obese patients with OSA to reduce missed diagnoses.

The prevalence of GERD among patients with OSA varies widely by case definition (symptom-based GERD vs. nocturnal reflux) and measurement method (questionnaire vs. objective testing). An OSA clinic series found 12.9% GERD diagnosed by questionnaire, which was similar to our findings [35]. Early identification of GERD in OSA is clinically important because reflux and sleep-disordered breathing share common risk factors and mutually aggravate arousal burden and autonomic activation. Undetected GERD contributes to fragmented sleep, chronic cough, laryngopharyngeal symptoms, dental erosion, and microaspiration, and it can undermine PAP tolerance through mouth breathing, aerophagia, and mask leak. Conversely, targeted GERD management improves nocturnal symptoms and quality of life and can enhance PAP adherence. Routine screening for reflux symptoms at OSA diagnosis can reduce misattribution of PAP side effects, and support integrated treatment plans that lower symptom burden and downstream respiratory complications. However, it should be noted that GERD questionnaires are mainly for screening and symptom tracking, not definitive diagnosis, and the accuracy for GERD questionnaires may be decreased for patients with OSA, because OSA can cause mouth breathing, xerostomia, chest discomfort, and aerophagia, mimicking reflux and inflating or obscuring scores. In OSA clinics, adding items on nocturnal/positional symptoms related to GERD and pursuing objective testing for persistent, atypical, or high-risk cases are important.

Previous studies found that comorbidity burden increased progressively with higher OSA severity, with severe OSA showing greater odds of resistant hypertension, atrial fibrillation, metabolic syndrome, and chronic kidney disease versus mild OSA [36,37]. Our current study found that nocturnal hypoxia (SPO_2_mean, ODI, or T90), rather than AHI, were associated with the number of total comorbidities and the prevalence of many cardio-metabolic complications, after adjustment for age, sex, BMI, smoking and systemic inflammation parameters. OSA severity has traditionally been defined by AHI; however, growing evidence suggests that AHI alone may insufficiently capture the pathophysiological burden of sleep-disordered breathing, particularly with respect to cardiometabolic risk. Some emerging metrics that quantify nocturnal hypoxemia, such as hypoxic burden may better reflect the cumulative hypoxic stress experienced during sleep. Recent large-scale observational studies have demonstrated that hypoxic burden is more strongly associated with adverse cardiometabolic outcomes than AHI [38,39]. These findings suggest that hypoxemia-related indices may capture clinically relevant aspects of OSA severity that are not adequately represented by AHI alone. Our results indicate that incorporating measures of oxygen desaturation may strengthen associations with comorbidity risk scales for OSA. Future studies should evaluate whether hypoxia-based metrics can better identify patients who derive the greatest benefit from targeted cardiometabolic screening and intervention. In addition, studies found that in patients with OSA, both objectively and subjectively measured sleep duration shows a nonlinear (U-shaped) association with cardiometabolic complications [40]. Compared with normal sleep duration, short sleep and long sleep are linked to higher risks of hypertension, type 2 diabetes, metabolic syndrome, and atherosclerotic events. In our relatively young cohort, we found that short sleep duration (<6h) was independently associated with increased risks of metabolic syndrome, HEpEF and NAFLD, which was in line with previous report. Therefore, integrating sleep duration and hypoxic metrics may improve comorbidity risk stratification and enhance prevention and treatment planning. Interestingly, the diagnostic yield of the structured comorbidity workup in our study was not associated with the severity of OSA, which may suggest that comorbidity screening is worthwhile across all OSA severity categories, as substantial diagnostic gains were also observed in mild and moderate cases.

Our findings reinforce the concept that OSA should be considered not merely as a disorder of sleep and breathing, but as a systemic condition frequently embedded within a network of cardiovascular, metabolic, respiratory, and neuropsychiatric comorbidities. Timely identification and management of these comorbidities is likely to prevents the “silent” damages, because these comorbidities may present for years without symptoms. The high proportion of patients in whom new diagnoses triggered changes in management in our study suggests that systematic comorbidity assessment could be integrated into routine OSA care pathways, ideally in multidisciplinary sleep-cardiology-endocrinology collaborations.

The strengths of our study include its real-world outpatient setting, the use of a standardised and comprehensive diagnostic protocol, and the focus on clinically actionable outcomes. The relatively large sample size also strengthens the precision of prevalence estimates. However, several limitations should be acknowledged. First, the absence of a contemporaneous “usual care” comparator precludes direct quantification of the incremental diagnostic value of the structured screening protocol relative to usual practice. Consequently, the diagnostic yield observed in this study should not be interpreted as evidence of superiority over routine care, but rather as an estimate of the burden of previously unrecognized comorbidities that can be identified when a standardized screening approach is applied in a real-world OSA clinic population. This design may introduce interpretation bias, as the observed yield reflects both true underlying comorbidity prevalence and the increased intensity and uniformity of ascertainment inherent to protocol-driven screening. Nonetheless, from a pragmatic clinical perspective, these findings remain informative, as they highlight the magnitude of potentially missed cardiometabolic comorbidities in routine practice and underscore the need for more systematic approaches to comorbidity assessment. Future prospective studies incorporating a usual-care comparator or stepped-wedge designs are warranted to directly quantify incremental diagnostic benefit and evaluate downstream clinical impact. Second, the sex distribution of the study population, with a predominance of male patients, limits the direct generalizability of our findings to female patients with OSA, although it reflects the sex distribution typically observed in real-world sleep clinic cohorts. The current findings should therefore be interpreted as most applicable to typical outpatient OSA populations, while underscoring the need for future studies specifically designed to evaluate sex-specific screening yield and to improve cardiometabolic risk detection in women with OSA. The single-centre design may also limit generalizability to other healthcare systems. Third, several conditions, including dyslipidemia and metabolic syndrome, were identified based on standardized laboratory or clinical measurements obtained during a single study visit. As a result, the reported prevalence may overestimate the proportion of patients who would ultimately meet criteria for a confirmed clinical diagnosis following repeated testing or longitudinal follow-up. Fourth, this cross-sectional screening study does not evaluate whether the identification of newly detected comorbidities leads to improvements in clinical outcomes, such as CPAP adherence, cardiometabolic risk factor control, or cardiovascular events. Diagnostic yield should therefore be interpreted as an indicator of unmet clinical need rather than as evidence of improved prognosis. Future prospective studies incorporating follow-up and outcome assessment are required to determine whether systematic screening, coupled with targeted intervention, translates into measurable improvements in patient-centered and cardiovascular outcomes. Finally, the comprehensive nature of the screening protocol used in this study also raises important considerations regarding resource utilization and cost-effectiveness. Whether this approach is feasible or cost-effective to implement uniformly across all patients with OSA in routine clinical practice deserves to be further investigated. Future studies incorporating health economic evaluations are needed to clarify whether selected components of this screening approach, potentially guided by clinical risk stratification, can achieve an optimal balance between diagnostic yield, cost, and clinical benefit.

## Conclusion

Our study demonstrates the substantial diagnostic yield of a structured comorbidity screening workup in patients with OSA, identifying a median of two previously unrecognized comorbidities per patient in addition to pre-existing conditions. Cardiometabolic complications were the most frequently unrecognized comorbidities. Furthermore, our findings indicate that these sub optimally treated comorbidities represent clinically actionable conditions. Using real-world data, our study proposes a comprehensive and pragmatic approach to routine comorbidity screening in patients with OSA. Integrating systematic comorbidity screening into OSA care may improve risk stratification, inform treatment decisions, and enhance patient treatment motivation, with potential benefits for both symptom control and the prevention of adverse cardiometabolic outcomes.

## Supplementary Material

Supplement flowchart.pdf

## Data Availability

The data that support the findings of this study are available from China Japan Friendship Hospital. But restrictions apply to the availability of these data, which were used under license for the current study, and so are not publicly available. Data are however available from the authors upon reasonable request and with permission of China Japan Friendship Hospital.
