# Supplementary figures and images for: The diagnostic yield of a structured comorbidity workup in a real-life outpatient population with obstructive sleep apnea: a cross-sectional study
Source: Ann Med. 2026 Feb 4;58(1):2622175. doi: 10.1080/07853890.2026.2622175 (PMC12879501; doi:10.1080/07853890.2026.2622175)

## Distribution of Baseline and Newly Detected Comorbidities by OSA Severity

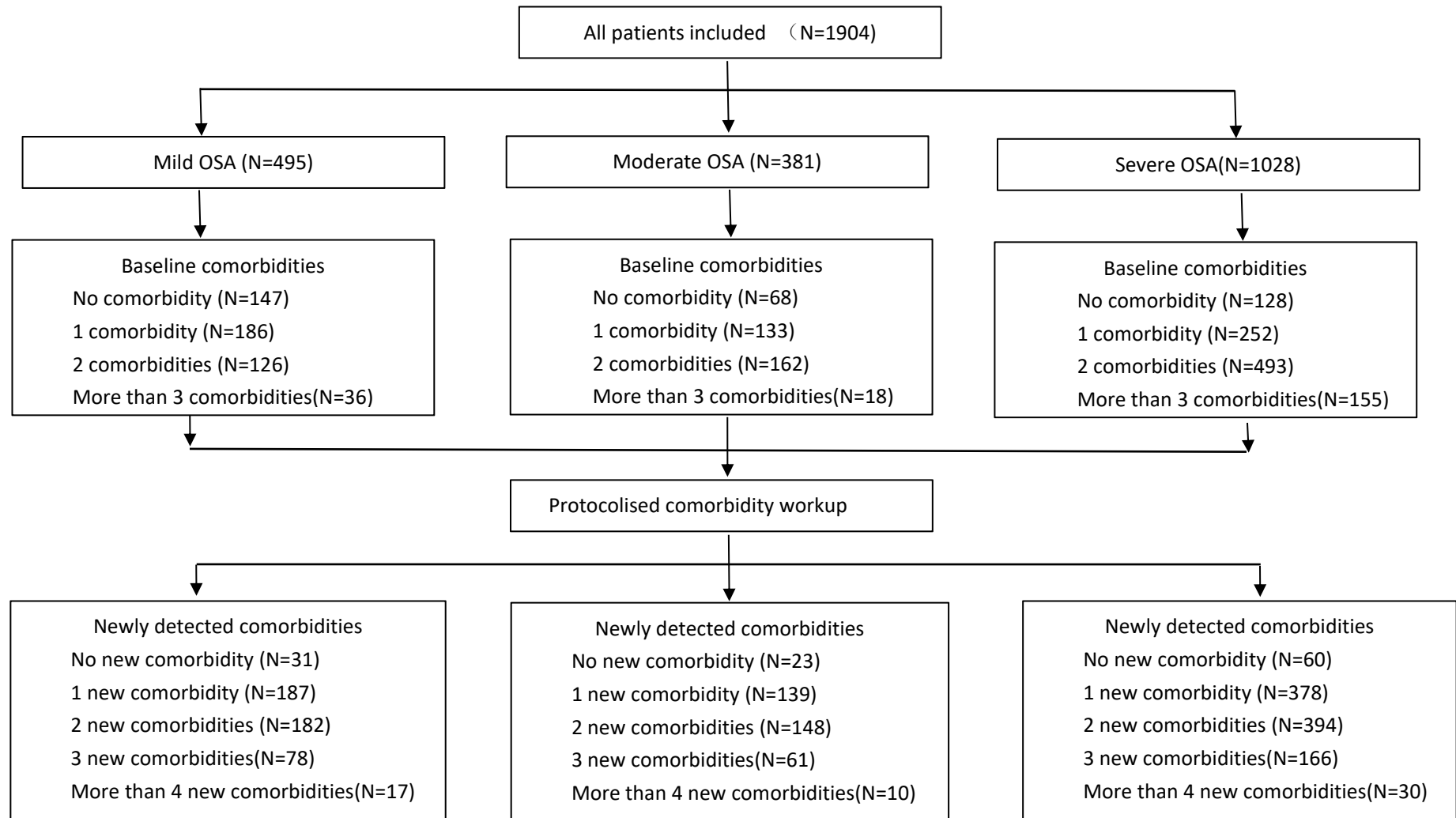

Supplement: Supplement flowchart.pdf [file IANN_A_2622175_SM3000.pdf]
